# Supplementary material for: Crop Proteomics under Abiotic Stress: From Data to Insights
Source: Plants (Basel). 2022 Oct 27;11(21):2877. doi: 10.3390/plants11212877 (PMC9657731; doi:10.3390/plants11212877)
Supplement: Supplementary file 1 [file plants-11-02877-s001.zip › plants-1937509-supplementary.pdf]

Table S1: Crop proteomics under abiotic stress during 2017-2022

Crop (stress)	Organ	Method	No*	Category	Major findings	Ref**
Rice (drought)	Flag leaf	LC/MS	18	Stomatal conductance, chlorophyll synthesis, HCR proteins	Synthetic cytokinin reversed stress induced alterations by decreasing chlorophyll synthase and HCR proteins.	Gujjar et al., 2020
	Leaf	LC-MS/MS	12	Photosynthesis, oxidative stress, stress signaling	Protein modulation occurred to cope with oxidative stress, maintaining photosynthesis and metabolism.	Auler et al., 2021
		LC-MS/MS	57	Metabolic, cellular, biological progress, stress response	OsSKIPa/ OsPP2C induced in transgenic line and prohibitin/ PsbP/ msrB accumulated.	Shi et al, 2018
		LC-MS/MS	213	Heat shock and late embryogenesis associated proteins	Mitochondrial import inner membrane translocase, Q10MK4, changed in all genotypes along with ribonuclease3.	Hamzelou et al., 2020
		iTRAQ LC-MS/MS	324	Abiotic stress, circadian rhythm, ROS	OsPYL9 mutagenesis conferred by increasing enzymatic activities and wax accumulation. GIGANTEA, adagio-like, and pseudo-response regulator proteins showed higher interaction in protein–protein interaction network.	Usman et al., 2020
	Root	LC-MS/MS	1138	Cellular oxidant detoxification, root elongation, iron sulfur cluster assembly	Oxidant detoxification, iron/sulfur cluster assembly system, ROS homeostasis, cell growth/elongation were changed in root tips of drought tolerant cultivar.	Abdirad et al, 2022
	Panicle/ Spike	iTRAQ LC/MS	244	Biosynthesis of secondary metabolites, metabolic pathways, amino acid, carbohydrate, energy metabolism, environmental adaptation, lipid metabolism	Photosynthesis process, energy metabolism and ROS metabolism were negatively affected.	Xiong et al., 2019
Wheat (salt)	Germinating seed	LC/MS/MS	397	Phenyl-propanoid biosynthesis, fatty acid metabolism, cysteine/ methionine metabolism, starch/sucrose metabolism	The phenyl-propanoid biosynthesis and fatty acid degradation pathways were enriched as the ubiquitous stress responses.	Yan et al., 2020
	Seedling	UPLC-MS	194	Calvin cycle, transcription and translation, amino acid metabolism, anti-oxidation, protein folding, proteolysis, carbon, nitrogen metabolism	Chloroplast proteins involved in light-dependent reaction decreased.	Zhu et al., 2021
	Root	Orbitrap LC-MS/MS	121	Stress/defense, carbohydrate, energy metabolism, transcription-related, transport, signaling, protein synthesis/degradation	Substrate-recruiting E3 ubiquitin ligases facilitated the modulation of key downstream stress-responsive transcription factors and BZIP-type transcription factor up-regulated.	Jiang et al., 2017

	Leaf/ Root	Label free LC-MS/MS	41	Late embryogenesis abundant proteins, dehydrin related, catalases, iso-citrate dehydrogenases	Malate dehydrogenase and malic enzyme were regarded as candidate sodic alkaline-tolerance proteins.	Han et al., 2019
	Leaf	EASY LC-MS/MS	234	Calcium transporting ATPases, chaperon protein folding/ quality control, stress responsive unfolded protein, endoplasmic reticulum related degradation	Pathways of UPR and ERAD and the synergistic response of endoplasmic related proteins played important roles in salt stress defense mechanism.	Zhang et al., 2021
Maize (Osmotic stress)	Seedling	iTRAQ-LC-MS/MS	270	Ribosomal structure and biogenesis, translation, posttranslational modification, protein turnover, chaperones	Protein biosynthesis, ROS scavenging, nitrogen and glutamate metabolism, energy homeostasis, and nucleic acid synthesis were positively correlatively for stress tolerance.	Weng et al., 2021
	Shoot	LTQ-Orbitrap UPLC	284	Photosynthesis, amino acid, sugar/ starch metabolism, redox-regulation	Starch biosynthesis contributed for the maintenance of leaf growth under drought stress and facilitated enhanced carbon acquisition upon recovery.	AbdElgawad et al., 2020
	Leaf	MALDI-TOF-TOF-MS	104	Photosynthesis, carbohydrate metabolism, stress defense, energy production, protein metabolism	The rates of translation and protein degradation differ among different proteins and the differences in ribosomal composition led to altered mRNA preference, which was responsible for the translation efficiency required for water-stress adaptation.	Xin et al., 2018
		UHPLC-MS/MS	1459	Cell wall biosynthesis, sugar/ amino acid metabolism, microtubule biosynthesis	Arabinose in cell wall-bound hemicellulose increased in abundance and stiffening of leaf cell wall occurred during salt stress.	Geilfus et al., 2017
		iTRAQ-LC-MS	300	Photosynthesis, antioxidants/ detoxifying enzymes, chaperones, metabolic enzymes	Proteins related to photosystem II repair and photoprotection improved photochemical capacity in tolerant cultivar.	Li et al., 2021
	Root	UPLC-MS/MS	1747	Oxidative stress, dehydration, respiration, translation, protein synthesis, ion compartmentalization	Lower concentrations of a potentially channel-regulating protein and higher abundance of proteins acting in protein synthesis and possible ion compartmentation with less ions in shoots were recorded in relatively salt stress tolerant genotype.	Soares et al., 2018
		iTRAQ-LC-MS/MS	513	Pentose phosphate pathway, glutathione metabolism, and nitrogen metabolism	Superoxide dismutase activity, malondialdehyde content, relative electrolyte leakage, and proline content were consistent with the predicted changes based on their functions.	Luo et al., 2018
		iTRAQ LC-MS/MS	7723	Ribosome pathway, glycolysis/gluconeogenesis pathway, amino sugar, nucleotide sugar metabolism	The promotion of enzymes in the glycolysis/gluconeogenesis pathway, cross-protection against the toxicity of aldehydes and ammonia and the maintenance of cell membrane stability	Zeng et al., 2019

					were the key mechanisms of tolerance in drought-tolerant variety.	
Soybean (flood)	Root	LC-MS/MS	351	Protein degradation, stress targeting, PTM, amino acid activation, protein folding, transport, cell wall, signaling, photosynthesis, ribosome	Calnexin/calreticulin and glycoproteins increased.	Hashimoto et al., 2020
	Root, hypocotyl	LC-MS/MS	15	Glycolysis, TCA cycle	Glyceraldehyde-3-phosphate dehydrogenase, aconitase 1, and 2-oxoglutarate dehydrogenase configured as flood responsive candidate set.	Wang et al., 2021
		LC-MS/MS	66	Photosynthesis, lipid metabolism, protein synthesis, redox, trehalose, mitoETC, glycolysis	Improved growth of soybean seedlings irradiated with millimeter waves was due to the up-regulation of APX, sugar metabolism, and redox related pathways.	Zhong, et al., 2020
		LC-MS/MS	169	Glycoproteins, endoplasmic reticulum proteins	Glycoproteins folding and accumulation of calnexin, calreticulin, alcohol dehydrogenase played an important role for the acquisition of flooding tolerance in mutant soybean.	Komatsu et al., 2021
		LC-MS/MS	274	Starch/sucrose metabolism, glycolysis	O-fucosyltransferase increased.	Li et al., 2018
	Root tip	HPLC-MS/MS	1401	Eukaryotic aspartyl protein, 13-hydroxylupanine O-tigloyl-transferase, RNA/cell wall metabolism	Melatonin treatment promoted soybean growth under flooding stress through the recovery of reduced lignification in cell wall and abundance of 13-hydroxylupanine O- tigloyl-transferase.	Wang et al., 2020
		LC-MS/MS	176	Photosynthesis, RNA, DNA, signaling, TCA cycle, carbohydrate metabolism	Calcium homeostasis played the bridging role between cytosol and subcellular compartments during flooding stress.	Wang et al., 2017
	Leaf, Root, hypocotyl	LC-MS/MS	157	Photosynthesis, RNA, DNA, signaling, TCA cycle	Beta-amylase 5 played a role in enhancing starch degradation to provide carbohydrate intermediates for cellular metabolisms in the leaf of soybean seedlings under flooding.	Wang et al, 2018

*No: Number of identified proteins; **Ref: Reference

Abbreviations:

HCR: chlorophyll synthase and 7-hydroxymethyl chlorophyll a reductase; GIGANTEA: GI of Arabidopsis involved in flowering time localized in nuclear plasma membrane; SKIPa, ski-interacting protein; PP2C, protein phosphatase class 2; PYL9, Pyrabactin resistance 1 like/ regulatory components of ABA receptor 9; UPR, unfolded protein response; ERAD, endoplasmic reticulum–associated protein degradation; ROS, reactive oxygen species; TCA, tricarboxylic acid cycle; PTM, post-translational modification; mitoETC, mitochondrial electron transport chain; APX, ascorbate peroxidase.

References:

AbdElgawad, H.; Avramova, V.; Baggerman, G.; Raemdonck, G. V.; Valkenborg, D.; Ostade, X. V.; Guisez, Y.; Prinsen, E.; Asard, H.; Ende, W. V.; Beemster, G. T. S. Starch biosynthesis contributes to the maintenance of photosynthesis and leaf growth under drought stress in maize. *Plant Cell Environ*. **2020**, *43*, 2254-2271.

Abdirad, S.; Wu, Y.; Ghorbanzadeh, Z.; Proteomic analysis of the meristematic root zone in contrasting genotypes reveals new insights in drought tolerance in rice [published online ahead of print. *Proteomics* **2022**, e2200100.

Auler, P. A.; Nogueira do-Amaral, M.; Bolacel Braga, E. J.; Maserti, B. Drought stress memory in rice guard cells: Proteome changes and genomic stability of DNA. *Plant Physiol. Biochem*. **2021**, *169*, 49-62.

Geilfus, C. M.; Tenhaken, R.; Carpentier, S. C. Transient alkalinization of the leaf apoplast stiffens the cell wall during onset of chloride salinity in corn leaves. *J. Biol. Chem*. **2017**, *292*, 18800-18813.

Gujjar, R. S.; Banyen, P.; Chuekong, W.; Worakan, P.; Roytrakul, S.; Supaibulwatana, K. A synthetic cytokinin improves photosynthesis in rice under drought stress by modulating the abundance of proteins related to stomatal conductance, chlorophyll contents, and rubisco activity. *Plants* **2020**, *9*, 1106.

Hamzelou, S.; Pascovici, D.; Kamath, K. S.; Amirkhani, A.; McKay, M.; Atwell, B. J.; Haynes, P. A.; Proteomic Responses to Drought Vary Widely Among Eight Diverse Genotypes of Rice (*Oryza sativa*). *Int. J. Mol. Sci*. **2020**, *21*, 363.

Han, L.; Xiao, C.; Xiao, B.; Wang, M.; Liu, J.; Bhanbhro, N.; Khan, A.; Wang, H.; Wang, H.; Yang, C. Proteomic profiling sheds light on alkali tolerance of common wheat (*Triticum aestivum* L.). *Plant Physiol. Biochem*. **2019**, *138*, 58-64.

Hashimoto, T.; Mustafa, G.; Nishiuchi, T.; Komatsu, S. Comparative Analysis of the Effect of Inorganic and Organic Chemicals with Silver Nanoparticles on Soybean under Flooding Stress. *Int. J. Mol. Sci*. **2020**, *21*, 1300.

Jiang, Q.; Li, X.; Niu, F.; Sun, X.; Hu, Z.; Zhang, H. iTRAQ-based quantitative proteomic analysis of wheat roots in response to salt stress. *Proteomics* **2017**, *17*, 10.1002

Komatsu, S.; Yamaguchi, H.; Hitachi, K.; Tsuchida, K.; Kono, Y.; Nishimura, M. Proteomic and Biochemical Analyses of the Mechanism of Tolerance in Mutant Soybean Responding to Flooding Stress. *Int. J. Mol. Sci*. **2021**, *22*, 9046.

Li, B.; Takahashi, D.; Kawamura, Y.; Uemura, M. Plasma membrane proteomics of Arabidopsis suspension-cultured cells associated with growth phase using nano-LC-MS/MS. *Methods Mol. Biol*. **2018**, *1696*, 185-194.

Li, H.; Yang, M.; Zhao, C.; Wang, Y.; Zhang, R. Physiological and proteomic analyses revealed the response mechanisms of two different drought-resistant maize varieties. *BMC Plant Biol*. **2021**, *21*, 1513.

Luo, M.; Zhao, Y.; Wang, Y.; Shi, Z.; Zhang, P.; Zhang, Y.; Song, W.; Zhao, J. Comparative proteomics of contrasting maize genotypes provides insights into salt-stress tolerance mechanisms. *J. Proteome Res*. **2018**, *17*, 141-153.

Shi, F.; Yang, X.; Zeng, H.; Guo, L.; Qiu, D. Label-free quantitative proteomic analysis revealed a positive effect of ectopic over-expression of PeaT1 from *Alternaria tenuissima* on rice (*Oryza sativa*) response to drought. *3 Biotech*. **2018**, *8*, 480.

Soares, A. L. C.; Geilfus, C. M.; Carpentier, S. C. Genotype-specific growth and proteomic responses of maize toward salt stress. *Front. Plant Sci*. **2018**, *9*, 661.

Sun, F.; Yu, H.; Qu, J.; Cao, Y.; Ding, L.; Feng, W.; Khalid, M. H. B.; Li, W.; Fu, F. Maize ZmBES1/BZR1-5 decreases ABA sensitivity and confers tolerance to osmotic stress in transgenic *Arabidopsis*. *Int. J. Mol. Sci*. **2020**, *21*, 996.

Usman, B.; Nawaz, G.; Zhao, N.; Liao, S.; Liu, Y.; Li, R. Precise editing of the *OsPYL9* gene by RNA-guided Cas9 nuclease confers enhanced drought tolerance and grain yield in rice (*Oryza sativa* L.) by regulating circadian rhythm and abiotic stress responsive proteins. *Int. J. Mol. Sci*. **2020**, *21*, 7854.

Wang, J.; Ren, Y.; Liu, X. Transcriptional activation and phosphorylation of OsCNGC9 confer enhanced chilling tolerance in rice. *Mol. Plant* **2021**, *14*, 315-329.

Wang, X.; Komatsu, S. Proteomic approaches to uncover the flooding and drought stress response mechanisms in soybean. *J. Proteomics* **2017**, *172*, 201-215.

Wang, X.; Li, F.; Chen, Z.; Yang, B.; Komatsu, S.; Zhou, S. Proteomic analysis reveals the effects of melatonin on soybean root tips under flooding stress. *J. Proteomics* **2020**, *232*, 104064.

Wang, X.; Sakata, K.; Komatsu, S. An integrated approach of proteomics and computational genetic modification effectiveness analysis to uncover the mechanisms of flood tolerance in soybeans. *Int. J. Mol. Sci*. **2018**, *19*, 1301.

Weng, Q.; Zhao, Y.; Yanan, Z.; Song, X.; Yuan, J.; Liu, Y. Identification of salt stress-responsive proteins in maize (*Zea may*) seedlings using iTRAQ-based proteomic technique. *Iran. J. Biotechnol*. **2021**, *19*, e2512.
Xin, L.; Zheng, H.; Yang, Z.; Guo, J.; Liu, T.; Sun, L.; Xiao, Y.; Yang, J.; Yang, Q.; Guo, L. Physiological and proteomic analysis of maize seedling response to water deficiency stress. *J. Plant Physiol*. **2018**, *228*, 29-38.
Xiong, Q.; Cao, C.; Shen, T.; Zhong, L.; He, H.; Chen, X. Comprehensive metabolomic and proteomic analysis in biochemical metabolic pathways of rice spikes under drought and submergence stress. *Biochim. Biophys Acta Proteins Proteom*. **2019**, *1867*, 237-247.
Yan, M.; Xue, C.; Xiong, Y.; Meng, X.; Li, B.; Shen, R.; Lan, P. Proteomic dissection of the similar and different responses of wheat to drought, salinity and submergence during seed germination. *J. Proteomics* **2020**, *220*, 103756.
Zeng, W.; Peng, Y.; Zhao, X.; Wu, B.; Chen, F.; Ren, B.; Zhuang, Z.; Gao, Q.; Ding, Y. Comparative proteomics analysis of the seedling root response of drought-sensitive and drought-tolerant maize varieties to drought stress. *Int. J. Mol. Sci*. **2019**, *20*, 2793.
Zhang, J.; Liu, D.; Zhu, D.; Liu, N.; Yan, Y. Endoplasmic reticulum subproteome analysis reveals underlying defense mechanisms of wheat seedling leaves under salt stress. *Int. J. Mol. Sci*. **2021**, *22*, 4840.
Zhong, Z.; Furuya, T.; Ueno, K.; Yamaguchi, H.; Hitachi, K.; Tsuchida, K.; Tani, M.; Tian, J.; Komatsu, S. Proteomic Analysis of Irradiation with Millimeter Waves on Soybean Growth under Flooding Conditions. *Int. J. Mol. Sci*. **2020**, *21*, 486.
Zhu, D.; Luo, F.; Zou, R.; Liu, J.; Yan, Y. Integrated physiological and chloroplast proteome analysis of wheat seedling leaves under salt and osmotic stresses. *J. Proteomics* **2021**, *234*, 104097.
